# PReS-FINAL-2135: Analysis of the HLA region in a large cohort of juvenile idiopathic arthritis cases identifies independent effects at HLA-DRB1

**DOI:** 10.1186/1546-0096-11-S2-P148

**Published:** 2013-12-05

**Authors:** A Hinks, J Cobb, B Han, MC Marion, M Sudman, P Martin, J Bohnsack, LR Wedderburn, JP Haas, PI De Bakker, CD Langefeld, S Raychaudhuri, S Prahalad, SD Thompson, W Thomson

**Affiliations:** 1 Arthritis research UK epidemiology unit, university of manchester, Manchester, UK; 2 Division of Genetics, Brigham and Women's Hospital, Harvard Medical School, Boston, MA, USA; 3 Department of Biostatistical Sciences, Wake Forest University School of Medicine, Winston-Salem, NC, USA; 4 Division of Rheumatology, Cincinnati Children's Hospital Medical Center, Cincinnati, USA; 5 School of Medicine, University of Utah, Salt Lake City, UT, USA; 6 Rheumatology Unit, UCL Institute of Child Health, London, UK; 7 German Centre for Rheumatology in Children and Young People, Garmisch-Partenkirchen, Germany; 8 Department of Pediatrics, Emory University School of Medicine, Atlanta, UK

## Introduction

Juvenile idiopathic arthritis (JIA) is the most common arthritic disease of childhood and is caused by a combination of genes and environment. In the last few years great advances have been made in dissecting the genetic basis of JIA with now 17 confirmed susceptibility loci identified. One of these loci, the MHC region, has been established for many years but the complexity and the broad linkage disequilibrium (LD) across the region has rendered fine-mapping associations challenging. Novel imputation strategies can now be utilized to impute HLA classical alleles and amino acids across the region.

## Objectives

The aim of this work was to gain a greater understanding of the associations across the HLA region in the 2 most common subtypes of JIA, oligoarthritis and rheumatoid factor-negative polyarthritis.

## Methods

Using the dense genotype data obtained from the analysis of the custom-designed Illumina immunochip in 2816 JIA cases and 13056 controls, we imputed HLA classical alleles (2-digit and 4-digit resolution) and amino acids across the MHC region (Chr6:29-34 Mb) using the SNP2HLA algorithm and a large reference panel of 5225 individuals from the T1DGC consortium. We performed logistic regression for all markers across the region and tested all amino acids in HLA-DRB1 performing an omnibus test of amino acid residues for each position. Conditional analysis to identify potential independent effects was performed.

## Results

We observed high correlation (0.99) between imputed and classically typed actual allele frequencies for HLA-DRB1 2-digit and 4-digit alleles for a subset of JIA cases (n = 394). The most significant association across the HLA region was for the phenylalanine residue at amino acid 67 of HLA-DRB1, OR = 3.03, p = 1 x 10^-179^. The omnibus test for all amino acids across HLA-DRB1 showed most significant association at HLA-DRB1 amino acid 13 and conditioning on all residues at amino acid 13 found significant association remaining at HLA-DRB1 amino acid 67, suggesting two independent effects in HLA-DRB1.

## Conclusion

Analysis of the MHC region in the largest cohort of JIA cases and controls studied to date has found the strongest association with the HLA-DRB1 region. Two independent effects have been identified, amino acid 67 and 13. Interestingly, amino acid 13 has previously been associated with adult rheumatoid arthritis (RA) whereas amino acid 67 has not. Further analysis to look for independent effects across the rest of the HLA region is now underway.

## Disclosure of interest

None declared.

